# Changing Times: New Possibilities for Community Health and Well-Being

**Published:** 2007-06-15

**Authors:** Leandris C Liburd, Joe Sniezek

**Affiliations:** Division of Adult and Community Health, National Center for Chronic Disease Prevention and Health Promotion, Centers for Disease Control and Prevention; Division of Adult and Community Health, National Center for Chronic Disease Prevention and Health Promotion, Centers for Disease Control and Prevention, Atlanta, Ga

Since antiquity, humankind has been concerned with disease and curing disease and with health and the conditions of health. Asclepius, the Greek hero who later became the Greek god of medicine and healing, used drugs to treat many diseases and taught others, including his children, about the healing arts. Two of Asclepius's daughters, Panacea and Hygeia, represent the two health models still in effect. Panacea (Greek for *all healing)* represents the medical model with its focus on curing disease. Hygeia (Greek for *health*) represents the model that focuses on preventing disease, maintaining health, and living rationally by exercising regularly, eating nutritional foods, and creating healthful living environments. The National Expert Panel on Community Health Promotion refocuses attention on the conditions of health consistent with Hygeia's view, attending not only to habits of living but also to the environment in which we live.

The National Center for Chronic Disease Prevention and Health Promotion (NCCDPHP), established in 1988, has a rich legacy of leadership in health promotion. The combined efforts of the 10 divisions and offices that make up NCCDPHP have produced an impressive portfolio of effective behavioral, lifestyle, and policy interventions intended to reduce the burden of chronic diseases in the United States. The National Expert Panel on Community Health Promotion was convened to review NCCDPHP's previous efforts and to suggest future directions. The work of the expert panel resulted in a call to CDC to broaden its view from a narrow focus on changing individual behavior to a wide focus on changing the social, political, and physical environments in which people live and make choices. These factors either facilitate or impede people's opportunity for a healthy and productive life.

Research data support the primary themes that emerged from the expert panel's deliberations (namely, the large effect of environments in shaping health outcomes; the power of income and education in providing opportunities for good health; and the necessary but insufficient public health focus on personal choice). As our knowledge of the historic, social, political, and economic determinants of chronic disease risk increases, we cannot ignore societal risk factors that, until recently, were considered outside the purview of public health and, therefore, were not central to recent chronic disease prevention efforts.

In the 19th and early 20th century, the leading cause of death was infectious disease. During this period, most improvements in health resulted from better nutrition, improved sanitation, and smaller family size (1). Success in controlling infectious disease was such that by the latter part of the 20th century, chronic disease had taken the lead as the major cause of mortality. Terris (2) and Breslow (3,4) consider the epidemiologic transition from infectious to chronic disease as the leading cause of death as the dividing line between two eras in public health, the first centered on controlling communicable diseases and the second, on controlling chronic diseases. Breslow (3,4), however, points ahead to a third era in which our approach is to maintain good health as a means of leading full and satisfying lives, a concept consistent with efforts to promote well-being. Breslow's approach is less reactive and less directed at problem solving (i.e., treating and preventing) and more forward looking than other approaches. From this point of view, health is more than merely the absence of disease; it is a resource that allows people to live full, productive, and satisfying lives. This perspective is aligned with the World Health Organization's definition of health as a state of complete physical, mental, and social well-being and not merely the absence of disease or infirmity (5).

**Figure:**
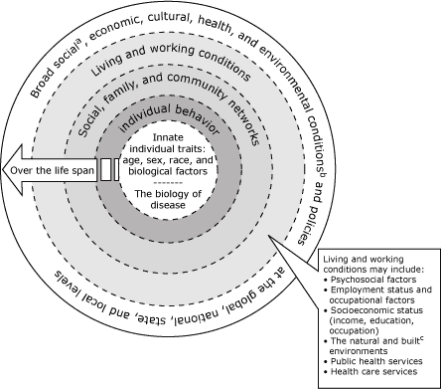
Institute of Medicine's model of the multiple determinants of health (6)

**Table:** 

a Social conditions include economic inequality, urbanization, mobility, cultural values, attitudes, and policies related to discrimination and intolerance on the basis of race, gender, and other differences.

b Other conditions at the national level include major sociopolitical shifts such as recession, war, and governmental collapse.

c The built environment includes transportation systems, water and sanitation systems, housing, and other dimensions of urban planning.

aSocial conditions include economic inequality, urbanization, mobility, cultural values, attitudes, and policies related to discrimination and intolerance on the basis of race, gender, and other differences.bOther conditions at the national level include major sociopolitical shifts such as recession, war, and governmental collapse.cThe built environment includes transportation systems, water and sanitation systems, housing, and other dimensions of urban planning.

Because the expert panel viewed health as a resource rather than simply as the absence of disease, panel members recommended new directions for community health promotion. These directions are aligned with the Institute of Medicine's (IOM) model (6) of the multiple determinants of health that illustrates the ecological nature of health and health status (Figure). Key components of the IOM model are also articulated in socioecological models of health promotion. For example, the model describes microlevel determinants (e.g., biological factors such as age and sex) at the center of a circle. These determinants interact with multiple outer layers that progressively comprise more macrolevel determinants (e.g., community networks, living conditions, large socio-political environments) to create conditions both within and external to the individual that tend toward positive and negative health outcomes. Overlying the social and physical circumstances encountered by individuals (e.g., protective social networks, unsafe and unsatisfying work conditions) in the outermost layer of the circle are global, national, and local economic and environmental policies that further affect health and the distribution of disease. The synergistic relationship among the multiple and complex layers of the circle makes it difficult to isolate any single component or risk factor embedded within the model as the principle driver of health status at any given point in time or over the course of the lifespan. Yet, we continue to expend a disproportionate amount of attention and resources in seeking change at the center of the circle through biomedical and behavioral interventions that largely ignore the macrolevel determinants of health.

The conventional and entrenched focus on individual behavior change is understandable given that public health activities related to chronic disease are often discussed in terms of primary, secondary, and tertiary prevention anchored at the individual level. Public health activities addressing chronic disease have often been classified according to disease or organ system (e.g., heart disease, arthritis, diabetes, cancer). The "medicalization" of public health by organ system tends to detract from efforts to promote the well-being of entire communities. In addition, health promotion is more than disease prevention: it encompasses both a reactive and proactive view of disease and well-being. In light of the contributions and perspectives presented in the invited editorials in this issue, we raise the question, Is the term *health promotion* adequate? Is it too closely identified with a key health promotion activity, health education? Does health promotion adequately relate the concept of promoting health and healthy social and physical environments to the public and public health professionals? As Lancaster and Anderson (7) point out in this issue, language is important: we need language that anchors health promotion to the contextual environment of health-related behavior. Although health promotion research grounds itself in the ecological framework and addresses social and physical environments, the term *health promotion* in public health practice may fail to convey the critical link or grounding between people's health and their environment.

Health promotion should focus on helping people thrive and not just on preventing them from getting sick. Health promotion within NCCDPHP should promote health as a resource that people use 1) to live full, productive, and satisfying lives and 2) to cope with or change unhealthful environments. Therefore, a renewed emphasis on affecting the outer rings of the IOM socioecologic model for health promotion (the social, family, and community networks; living and working conditions; and broad social, economic, cultural, health and environmental conditions), as recommended by the expert panel, will inspire public health workers and policy makers to think upstream and long term about ensuring the conditions that promote and protect health as a resource for life.

Responding to the recommendations of the expert panel is a daunting challenge to the current institutional structure, to epistemology as it applies to health promotion, and to the practice of public health in the United States. The recommendations may at first appear to be academic, pie-in-the-sky, and ignorant of the state-by-state, community-by-community governance of public health across the United States. In other words, the current system of public health in the United States does not have the resources, the practice paradigm, the workforce, the institutional facility, or the political will to take on issues embedded in structures over which we have little or no control. Tucker and Navarro (8) speak to both the opportunities and disincentives associated with several of the panel's recommendations. For example, the expert panel recommends community-based participatory research as an ideal method for engaging community members in identifying and addressing their health problems. However, the recommendation ignores past abuses that communities have experienced at the hands of researchers and perpetuates research that is driven more by the availability of funding and the interests of researchers than by the needs of the community. As we move toward expanded models of community health promotion practice, we must understand the historical public health and research experiences of a community. We must build trust and confidence in public health research among communities that experienced little benefit from past research. Indeed, often such research benefited the researcher more than the people studied.

As we plan the future of chronic disease prevention and community well-being, how can the expert panel's recommendations inform next steps and systems changes that will put us on the path to better health outcomes?  Lancaster and Anderson (7) argue that among our first tasks in moving toward greater community well-being is shortening the time between discovery of innovations and their use in community practice. To accelerate the translation of research into practice, we must rethink and refine our systems of accountability and our models for dissemination. Moreover, the time frames within which we conduct intervention research and implement programs must keep pace with the changing landscape and demographics of contemporary communities. Establishing frameworks for greater collaboration across categorical programs and among partners, as well as streamlining the steps in the research translation process, are actions within the control of NCCDPHP programs.

Historically policymakers and public health workers vacillated between the pros and cons of flexible funding (i.e., funding for research that is not disease-specific, is cross-cutting, and is open-ended for community input and direction rather than funding for projects that address a particular health condition). Once again, the expert panel calls for greater flexibility in the investment of federal resources that support community health promotion. In our current policy structure, funding of priority health concerns is influenced in large part by citizens and special interest groups who effectively argue their case with legislators for state and federal appropriations. If community health promotion is to thrive at the policy level and garner the greater financial support that will allow states and local communities the flexibility they need to invest in implementing the recommendations outlined by the expert panel, we must first galvanize a broader group of partners. Such partners would represent many sectors, including representatives from departments of education; businesses; housing and urban development agencies; and private, nonprofit, community-based organizations. Adamson and colleagues (9), representing the YMCA-USA and REACH (Racial and Ethnic Approaches to Community Health) 2010, address the inefficiency of chronic disease silos (separate entities that do not interact with each other) and the fragmentation of effort and overall inefficiency that can make connecting difficult at the local level. Efforts of the YMCA-USA and REACH 2010 have accelerated the pace of change and documented impressive improvements in health outcomes by funding teams of local leaders to direct change efforts.

The expert panel and several of this issue's authors — Freudenberg (10), Wilson and Satterfield (11), and Boyce and colleagues (12) — emphasize the need to have a public health workforce with the knowledge, skills, and tools necessary to effectively implement community health promotion, and a workforce that understands and is able to address the social determinants of health at the community level. Interventionists may need new skills to change their focus from people at high risk to the conditions that create risk. Boyce et al (12) and Tucker and Navarro (8) point out the need for a workforce that knows the nuances of *how* to do community level work. Siegel (13) shows us, for example, that only half of the states have the minimal recommended chronic disease epidemiologic workforce. Concerted efforts are needed to identify critical knowledge and skills necessary to measure, implement, and evaluate community public health programs. In addition, public health needs to include sociologists, anthropologists, political scientists, economists, and community psychologists in its workforce. Each field brings a new and unique perspective to public health problems. The consequences of the failure of public health to address the social context and its influence on health across the lifespan is passionately argued by Edelman (14) in her editorial on the cradle-to-prison pipeline that is robbing Latino and African American youth of full and satisfying lives.

The expert panel's recommendations to move beyond individual behavior change to a focus on improving living conditions across the lifespan will present challenges to existing programs. Lancaster and Anderson (7) point out that we need to identify areas where integration can happen, but we must also keep in mind the constraints of current program priorities, funding streams, and organizational structures at the federal, state, and local levels. In other words, as we expand our view of public health, we must ensure that we do no harm to core public health programs.

The leadership challenges that the panel's recommendations present are not without risks. Implementing recommendations will require resources that will perhaps be taken away from existing programs. We will need to decide whether we can move forward with varying levels of credible evidence. Because health-related program outcomes may not be realized for decades, intermediate program outcomes will need to be clearly defined. We will need to move forward with new skills and new partners in a new paradigm.

Many changes in health status in the late 19th and early 20th centuries resulted from changes in critical social and environmental determinants of health. The expert panel recommends that we once again focus our efforts on these determinants. If we are to effectively address the complex burden of chronic disease today, then we must change the practice paradigms that structure community health promotion and well-being. The work of the National Expert Panel on Community Health Promotion offers a roadmap to those new possibilities.
